# Kambo Frog Poison as a Cause of Esophageal Rupture

**DOI:** 10.7759/cureus.10677

**Published:** 2020-09-27

**Authors:** Ernesto S Robalino Gonzaga, Maria Chamorro, Latha Ganti, Robert Schneider

**Affiliations:** 1 Internal Medicine, University of Central Florida College of Medicine, Orlando, USA; 2 Emergency Medicine, University of Central Florida College of Medicine, Orlando, USA; 3 Emergency Medicine, Envision Physician Services, Plantation, USA; 4 Emergency Medical Services, Polk County Fire Rescue, Bartow, USA

**Keywords:** esophageal rupture

## Abstract

The authors present a case of a patient who used Kambo frog poison for body cleansing that induced severe vomiting and led to esophageal rupture followed by tension pneumothorax and septic shock. Kambo is the waxy substance secreted by the nocturnal giant tree frog *Phyllomedusa bicolor*. Kambo, which is poisonous, is commonly believed in South America to have cleansing and healing properties. As alternative medicine becomes more common, and as more tourists frequent our hospitals, knowledge of these types of ritual related exposures is important for the practicing emergency physician to be aware of.

## Introduction

Esophageal rupture is an uncommon pathology but carries high mortality and morbidity if not recognized early and managed appropriately [1,2]. Although rupture can occur in even a healthy individual, damaged epithelium and increased intra-esophageal pressure make the pathology more likely. Outcomes largely depend on the extent of rupture, intra-thoracic complications, and timeliness of interventions. The case below highlights an interesting agent, Kambo, which is a frog poison obtained from the secretions of the Amazon tree frog, that has been used for “ritual cleansing.” However, it can induce vomiting that increases intra-esophageal pressure and lead to rupture.

## Case presentation

**Figure 1:**
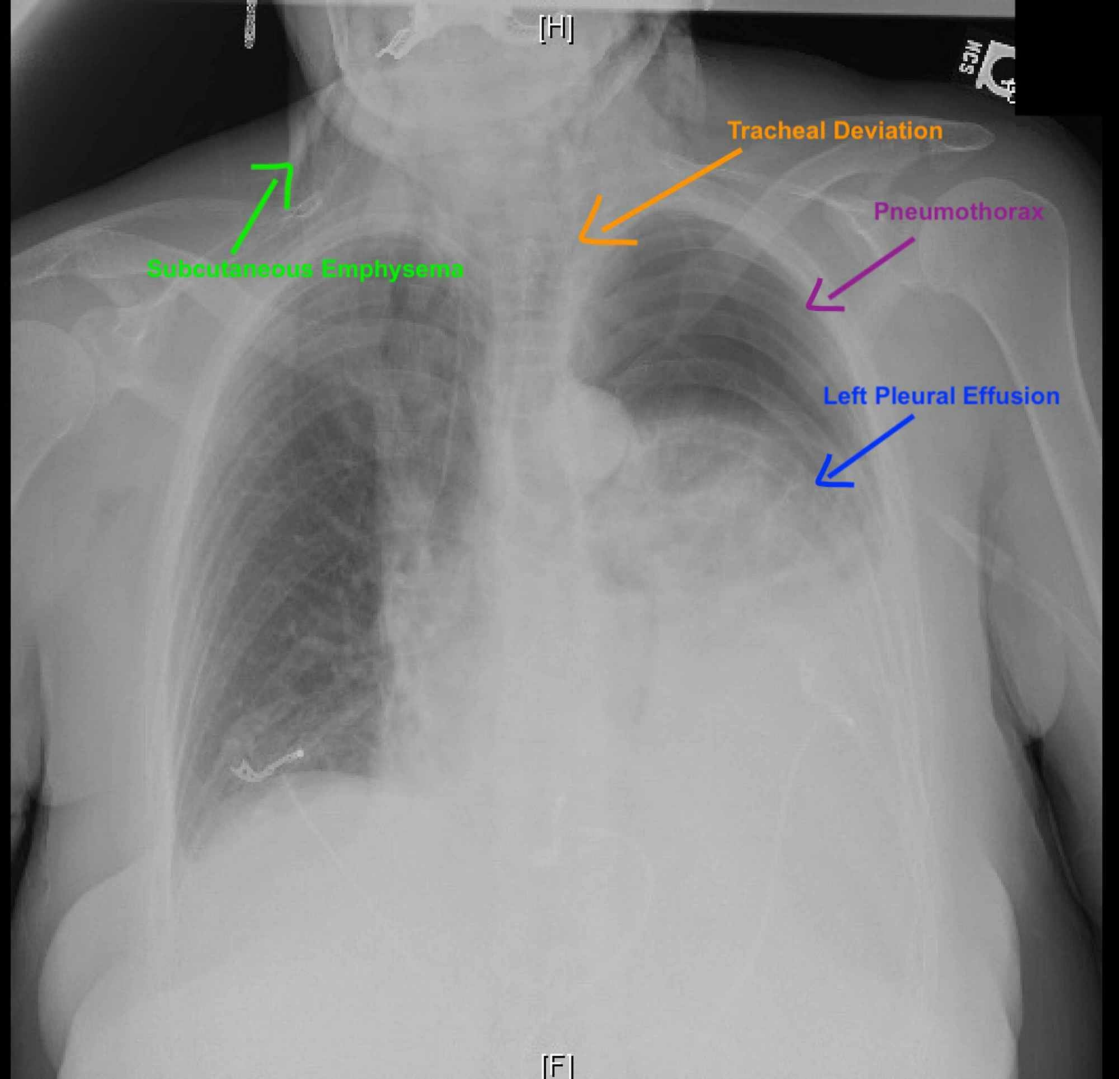
Chest radiograph demonstrating pneumothorax, pleural effusion, tracheal deviation, and subcutaneous emphysema

A 62-year-old woman with a past medical history of major depressive disorder, alcohol, and nicotine dependence presented to the emergency department with shortness of breath, epigastric abdominal pain, nausea, and non-bloody emesis for one day after using “Kambo” frog poison cream for body cleansing. Physical exam was relevant for tachycardia and oxygen saturation 95% on room air. She exhibited decreased breath sounds on auscultation and minimal crepitus on palpation near the base of the neck bilaterally, mild epigastric tenderness, and a soft abdomen with normal bowel sounds, no guarding or rebound. Laboratory evaluation showed leukocytosis with white blood cell count of 17 k/mm^3^, hemoglobin of 15 g/dl, lactic acidosis, and normal amylase and lipase. Chest radiograph was concerning for left-sided moderate sized tension pneumothorax and pneumomediastinum (Figure 1).

**Figure 2:**
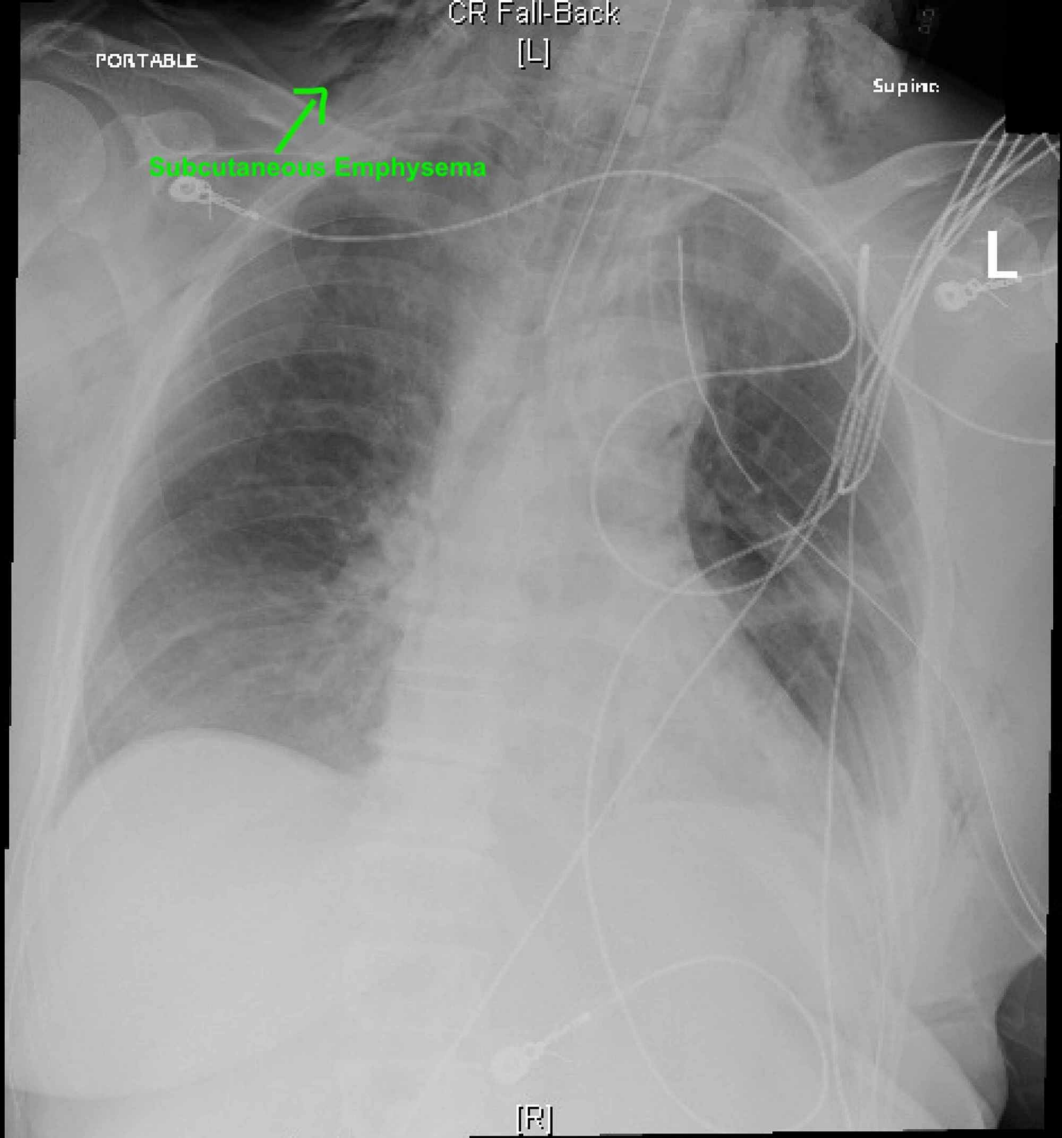
Chest radiograph post endotracheal intubation

CT chest and abdomen with IV contrast showed a large left-sided tension pneumothorax and a large left pleural effusion, and hyperdense material throughout the gastrointestinal tract suggesting of perforated esophagus. A left-sided chest tube was placed, and intubation for airway protection was performed (Figure 2).

The patient underwent esophageal repair via left thoracotomy and primary repair of esophageal perforation with placement of intracostal muscle flap.

## Discussion

Esophageal rupture occurs under conditions that increase intraesophageal pressure, including severe straining, vomiting, seizures, childbirth, prolonged coughing, or weightlifting. Additionally, conditions such as eosinophilic esophagitis, Barrett’s esophagus, strictures, or ulcerations can damage the esophageal mucosa and increase risk for rupture [2-5]. Kambo frog poison is obtained from the secretions of the Amazon tree frog, *Phyllomedusa bicolor*, that has been used as a traditional ritual for body cleansing. It contains opioid peptides and phyllocaerulein, a vasoactive molecule that induces intense vomiting. Other peptides found within the agent are sauvagine, a vasoactive molecule that can lead to hypotension, tachycardia, and diarrhea; phyllomedusin, a tachykinin peptide that can cause vasodilation and smooth muscle contraction and modify endocrine and exocrine secretory processes; and phyllokinin which relaxes arterial smooth muscle by acting on bradykinin receptors. In combination, these agents can increase risk of rupture through increased intra-esophageal pressure against the negative intra-thoracic pressure. There is little medical evidence regarding Kambo’s medicinal use while its use carries great risks, including sudden death [6,7]. There have been several case reports describing adverse events associated with Kambo use. In one case, a 44-year-old woman developed transient syndrome of inappropriate antidiuretic hormone after drinking six liters of water after a Kambo ritual [8]. An overweight man with a history of coronary disease was found dead after using Kambo. It is believed the cause of his death can be attributed to the vasoactive component that caused hypotension and tachycardia, leading to reduced myocardial perfusion. As is evident, there are a number of consequences that have been reported from the use of Kambo. However, no other reports of esophageal rupture have been made. Ruptures can lead to multiple complications, such as mediastinitis due to gastric content leaking into the mediastinal spaces, bacterial infections leading to mediastinal necrosis, pleural effusions, cardiac tamponade, sepsis, and organ failure [1,2,9]. Management of this pathology depends on hemodynamic status of the patient and location of the perforation. Conservative management can be considered in hemodynamically stable patients with contained perforation within the neck, mediastinum, or between the mediastinum and visceral lung pleura in non-neoplastic tissue injury, or proximal to an obstruction. Unstable patients or those with evidence of diffuse extravasation at site of perforation, progression of pneumomediastinum or pneumothorax, or development of empyema require immediate surgical intervention via open thoracotomy [4,10]. Endoscopic management can be considered for patients with high-risk surgical intervention [10]. Multiple techniques including stent placement, stent with endoscopic suturing, or endoluminal vacuum therapy can be utilized [11].

## Conclusions

This is a case of a female who used Kambo frog poison for body cleansing that induced severe vomiting and led to tension pneumothorax, septic shock, and esophageal rupture.
